# Correction: Evaluation of CRP as a marker for bacterial infection and malaria in febrile children at the Douala Gyneco-Obstetric and Pediatric Hospital

**DOI:** 10.1371/journal.pone.0334450

**Published:** 2025-10-14

**Authors:** The PLOS One Editors

## Notice of Republication

This article [1] was republished on August 7, 2025, to address an issue identified post-publication. An updated version of the S1 Database file is provided with this notice. Please download this article again to view the correct version.

The article’s Data Availability statement is updated to: The full data set has been uploaded as part of the submission, in Supporting Information files.

## Supporting information

S1 Database (XLSX)
