# Supplementary material for: Evaluation of CRP as a marker for bacterial infection and malaria in febrile children at the Douala Gyneco-Obstetric and Pediatric Hospital
Source: PLoS One. 2023 Jul 21;18(7):e0289012. doi: 10.1371/journal.pone.0289012 (PMC10361518; doi:10.1371/journal.pone.0289012)
Supplement: Attachment — Submitted filename: RESPONSE TO REVIEWERS.docx [file pone.0289012.s002.docx]

**RESPONSE TO REVIEWERS**

**RESPONSE TO ACADEMIC EDITOR**

1. The manuscript has been formatted to align with the journal’s style requirements.

2. I explained in my ethics statements that all participants (or their parents/legal guardians- this was a pediatric study) signed a consent form, meaning they gave written consent. I am confused as to how to clarify this further. However, I have highlighted the necessary line in the ethics statement, pending further clarifications.

3. I will be uploading my full data set with the revised manuscript as a supporting file.

**RESPONSE TO REVIEWER 1**

1. This was a formatting error. I have updated the manuscript with decimal points placed properly throughout.

2. I have replaced all sentences in the main text which began with numbers.

3. I have changed the line in the abstract to a more appropriate phrase.

4. Indeed, the study period was from the 15^th^ November 2020 to the 15^th^ of May 2021. It was a typing error which has been corrected.

5. The wording in the Discussion section has been revised.

**RESPONSE TO REVIEWER 2**

**Major points**

1. I have tried to reduce the spin I unfortunately put on the interpretation of predictive values. I’ve tried to take time to clarify how much they depend on the prevalence, and the possible pitfalls in comparing markers across studies. I do believe, however, that the predictive values in this study can be cautiously applied to most malaria-endemic areas, especially the NPV, for excluding malaria.

2. I did calculate positive and negative likelihood ratios based on different CRP thresholds, but I was unable to find other diagnostic studies for CRP and malaria which used them, so I was not able to properly discuss them. The closest I got was in Professor Lubell’s Cambodian study (reference 27), but he only did a positive likelihood ratio at 10mg/l and a negative likelihood ratio at 20mg/l, which makes it a bit difficult to compare with my study. However, I am willing to explore this further if the reviewer would clarify this point for me.

**Minor points**

1. The duration of fever included in the study was from 1 to 14 days. I have changed the symbol in the relevant section.

2. The radius of the BIOBASE centrifuge used was 9.50cm, approximating to 10. With the RPM at 3000, this gives a g value of 1006. I have changed the relevant section in the manuscript.

3. Indeed, the word was meant to be ‘instructions’ and not ‘instruments.’ That was a typo. The relevant section has been modified.

4. We used the prevalence of fever among pediatric consultations in a similar study by Mahende and co. in Tanzania, to determine the number of febrile children to sample. I have made the necessary precisions in the statistics section.

5. Given that CRP analysis has a wide range of numerical values, we used Youden’s J and ROC curves to determine which CRP value to use to determine the sensitivity and specificity for the different types of infection, as well as calculate predictive values. I have expanded on this explanation in the statistical analysis section of the revised manuscript.

6. TPF/μl refers to Trophozoites of Plasmodium Falciparum per microliter, which is the unit most commonly used to grade parasitemia. It is a measure of how many parasites are counted in each high-powered field on light microscopy multiplied by the number of red blood cells in each microliter of blood. I have added the definition to the manuscript.

7. The values in parentheses represent the 25% and 75% distribution intervals for the means. The values put before were erroneous. I apologize for the error. They were from an earlier data set that I forgot to update.

8. You are absolutely right. I have removed the phrases citing gold standard PPV and NPV values.

9. The reference is incorrect. I meant reference [27], Professor Lubell’s study on biomarker use in infectious states in certain southeastern Asian countries. I have corrected this error in the revised manuscript.
