# Supplementary material for: Evaluation of CRP as a marker for bacterial infection and malaria in febrile children at the Douala Gyneco-Obstetric and Pediatric Hospital
Source: PLoS One. 2023 Jul 21;18(7):e0289012. doi: 10.1371/journal.pone.0289012 (PMC10361518; doi:10.1371/journal.pone.0289012)
Supplement: Attachment — Submitted filename: RESPONSE TO REVIEWERS 3.docx [file pone.0289012.s003.docx]

**RESPONSE TO REVIEWERS**

**RESPONSE TO ACADEMIC EDITOR**

1. The reference list has been reviewed. It is complete and correct. All papers cited have been re-verified by searching on the PMC website. All textbooks cited have been verified to still be in circulation, and all websites cited have been checked to ensure they are still working and up-to-date.

**RESPONSE TO REVIEWER 1**

Thank you very much for your contributions to this work.

**RESPONSE TO REVIEWER 2**

I will begin by copying here the detailed responses to your previous round of reviews, since you mentioned you had not gotten them. The old points are highlighted in blue, while the ones for the most recent round of reviews are in normal print.

**Major points**

1. Thank you for your question regarding sample size calculation. The authors decided to use fever as the population determinant because we were investigating two different elements with differing prevalence in the pilot study. Malaria, for example, had a prevalence of 8.1% in Dr Mahende’s study, which translates to a minimal sample size of 113 children with Cochran’s formula. Bacterial infection, on the other hand, was found to be at 13% in the pilot study, giving a minimal sample size of 173 children. Since the admitting element was to be febrile children, we decided to use the prevalence of fever to calculate the sample size, especially since the value gotten (196 children) was safely above the minimal value for malaria from the pilot study, while bringing us closer to the values of malaria prevalence in Africa in literature (15-20%). However, I appreciate that this might have been a bit misleading, and am willing to detail this rationale in the article if you deem it necessary. The confidence interval mentioned was for the expected positive predictive values of malaria and bacterial infection after the optimal cut-offs. This is not necessary to include at that point, I think, so I have removed it to avoid further confusion.

2. Thank you for your insight. The values given before were written as 25% and 75% on the Epi Info dashboard, and I’m ashamed to say I wrongly represented these as 25^th^ and 75^th^ confidence intervals. They are, as you said, the 25^th^ and 75^th^ percentiles. I have replaced those values with the minimum and maximum values instead, as I believe those are more appropriate when discussing means.

3. Yes, it is indeed 23/29. I have changed the relevant section in the manuscript.

4. Dear sir/madam, thank you very much for this excellent point. I went back and checked the values, and this led me to some major modifications.

The first group of modifications concerns the data. There were some slight errors when entering into the software. Three patients in the earlier set had neither malaria, bacterial infection or viral infection, while four had both bacterial and viral infections (impossible considering the definition of viral infection in this study- that is, viral infections were considered only in those with neither proven malaria nor bacterial infection). I re-checked the electronic data using the physical questionnaires and have corrected these errors in the new data set I will be uploading with the revised manuscript.

The second group of modifications pertains to the various groups. Something to note- for clinical purposes those with malaria and viral infections were considered in the malaria group, so only those with viral monoinfection were considered in the ‘negative’ group. Also, those with both malaria and bacterial infections were counted both in the malaria and bacterial infections groups, as statistically they both would test ‘positive’ for the various infection types. The earlier analysis was done by a statistician who did not understand this. Most of the changes, therefore, will have to do with the number of false positives and thus the positive predictive ratios.

I redid the analysis myself, and will be uploading the positive and negative value tables as well as the new ROC curves in the revised manuscript. This has changed quite a few things in the article, as you will note. Again, thank you immensely for pointing out this discrepancy.

**Minor points**

1. I have changed the relevant sentence in the introduction.

**Major Points**

Dear Dr Geater, thank you again for your valuable contributions to this work. To address the issue of double infections, let me begin by saying that all the mixed infection cases were considered in the study. For the positive cases, all 50 cases of malaria were considered, as you will note- the 29 malaria monoinfections, the 14 cases of malaria and viral infection, and the 7 cases of malaria and bacterial infections. The same was done for the 49 cases of bacterial infection (here the two groups were bacterial monoinfection and malaria plus bacterial infection, as viral infections in this study were defined as febrile children who had been proven to have neither malaria nor bacterial infection). Double infections *were* considered in the positive cases. I couldn’t not consider them, because honestly many children present in the ER with malaria and viral rhinitis, and a significant number present with *both* malaria and enteric fever.

However, the first table discussing CRP distribution in the various groups is primordial, I believe, in guiding the definitions of positive and negative cases used to establish the ROC curves. Absolutely *no children* with both malaria and bacterial infections had low CRP values. In fact, they had the highest CRP values in the study. Notice also, that globally children with both malaria and viral infections actually had higher CRP values than those with malaria monoinfection. The double infections were all considered (I believe, in fact, that this is one of the stronger points of this study, as none of the others I could find did this), and their impacts on the CRP levels were clearly shown (if not, perhaps, thoroughly discussed in the manuscript).

The viral monoinfection group, therefore, emerges as the best ‘control’ or ‘negative’ group, as all the mixed infections have shown that the predominating element, if you would, is the one that raises the CRP (that is, malaria or bacterial infection). Any child presenting with such mixed infection, therefore, can be reasonably assumed to have a high CRP value corresponding to the more serious infection. That is why I considered only viral monoinfections as the negative cases. The ROC curves and predictive values thus gotten show that values below the optimum cut-off are likely to have *only* viral infections, and thus do not need antimalarials or antibiotics. Those with values above the cut-offs orient to *any* of the positive cases- that is, they could have malaria only, bacterial infection only, malaria and bacterial infection, or malaria and viral infection. This is a large group, but once viral monoinfection is definitely excluded, distinguishing between them is fairly easy even in a low-income setting.

I have added a small paragraph explaining this as succinctly as possible in the manuscript.

**Minor Points**

1. I have added the units of CRP to the first sentence of the CRP Levels section.

2. I actually included the full set of data. I just had certain filters on in the Excel spreadsheet, as I had been analyzing the data with MedCalc for the new ROC curves. It is possible to see all the data by removing the filters from the CRP, bacterial infection and malaria columns. However, I will also be resending a version of the datasheet with no filters, where all 220 participants are readily visible.
